# Photocatalytic Performance of g-C_3_N_4_ for Organic Peroxide Production Wastewater Under Visible Light

**DOI:** 10.3390/molecules31122119

**Published:** 2026-06-16

**Authors:** Zichun Yan, Banban Qiang, Wankai Yan, Hongfu Li, Hao Zhang

**Affiliations:** 1 School of Environmental and Municipal Engineering, Lanzhou Jiaotong University, Lanzhou 730070, China; qiangbanban2026@outlook.com (B.Q.); zhanghao20261@outlook.com (H.Z.); 2 Key Laboratory of Yellow River Water Environment of Gansu Province, Lanzhou 730070, China; 3 Wuwei Housing Construction Apartment Section China Railway Lanzhou Group Co., Ltd., Wuwei 733000, China; yanwaikai2002@outlook.com

**Keywords:** organic peroxide production wastewater, photocatalytic oxidation, g-C_3_N_4_, visible light

## Abstract

To explore the treatment-efficient photocatalytic system for organic peroxide production wastewater under visible light, the g-C_3_N_4_ catalyst, synthesized via thermal polycondensation, exhibited distinct optical absorption properties confirmed by UV-vis diffuse reflectance spectroscopy (UV–vis DRS). Operational parameters—specifically pH, catalyst loading, light intensity, and reaction time—were systematically optimized. Under optimal conditions (pH 5, g-C_3_N_4_ dosage 1.0 g/L, light intensity 1300 W/m^2^, reaction time 4 h), the system removed 72.8% of the COD, significantly enhancing the wastewater biodegradability (B/C ratio increased from 0.118 to 0.193). Analytical techniques, including gas chromatography–mass spectrometry (GC-MS) and UV-vis absorption spectroscopy, verified the effective decomposition of organic contaminants. Furthermore, radical quenching assays identified superoxide radicals (·O_2_^−^) and photogenerated electrons (e^−^) as the primary reactive species driving the photocatalytic process, highlighting the potential of g-C_3_N_4_ for industrial wastewater pretreatment.

## 1. Introduction

Organic peroxides are compounds in which the hydrogen atoms of hydrogen peroxide are substituted by alkyl, aryl, and other organic groups. They are widely used as polymerization initiators [1], crosslinking agents [2,3,4], and curing agents [5]. However, the wastewater generated during their production is characterized by high organic concentration, strong irritating odor, poor biodegradability, and resistance to degradation [6,7]. These properties make such wastewater difficult to treat directly by biological processes, so pretreatment is essential to remove part of the refractory organic matter and improve its biodegradability [8,9,10]. Common pretreatment technologies for wastewater include Fenton oxidation [11], potassium persulfate oxidation [12], catalytic ozonation [13], and iron–carbon micro-electrolysis [14]. Furthermore, photocatalytic technology has gradually become an important approach in environmental pollution remediation due to its outstanding pollutant degradation performance and wide application scenarios [15,16,17,18].

In recent years, photocatalytic technology has exhibited excellent application potential in the treatment of refractory organic wastewater. Priyadarshini et al. successfully synthesized a visible-light-driven magnetic catalyst NiFe_2_O_4_@ZIF-67 for the photocatalytic treatment of ciprofloxacin under visible light. Within 60 min, the removal percentage was improved from 53% to 88%, demonstrating an excellent removal performance [19]. Kim et al. synthesized a ternary metal N_2_C_4_ ZIF photocatalyst that exhibited excellent organic degradation performance, achieving a 95.1% degradation rate for methylene blue. The catalyst could also effectively decompose melanoidin pigments in coffee and wastewater [20]. Yan et al. utilized iron–carbon micro-electrolysis to treat organic peroxide production wastewater. Under the optimal conditions of an iron filing dosage of 30.5 g/L, an Fe-C mass ratio of 1.01, a reaction time of 122.8 min, and an initial pH of 3.1, a COD removal percentage of 35.7% was achieved [21]. Ali Rıza Dinçer et al. utilized Fenton oxidation for the treatment of wastewater containing organic peroxides, and the results indicated its effectiveness in removing COD [22]. Nevertheless, research on visible-light photocatalytic oxidation for organic peroxide wastewater remains rarely reported. To date, only Li et al. from our group have synthesized CuTiO_3_ for the photocatalytic treatment of this wastewater. Under the optimal conditions of pH 5, a catalyst dosage of 1.2 g/L, a light intensity of 1300 W/m^2^, and a reaction time of 4 h, a COD removal percentage of 58.3% was achieved [23]. However, the preparation process of this catalyst is complex, which limits its engineering application. Therefore, the development of visible-light-responsive photocatalysts with high stability and rate is crucial for promoting the application of photocatalytic technology in the treatment of organic peroxide wastewater. Furthermore, the development of stable visible-light-driven photocatalysts has long been a research hotspot [24,25,26,27].

As a typical metal-free polymeric semiconductor, g-C_3_N_4_ is prepared via thermal polycondensation of precursors such as melamine and urea. With melamine rings or triazine rings as the basic units, it forms a graphite-like layered conjugated π-electron system through C-N covalent bonds. g-C_3_N_4_ possesses advantages, including an appropriate band gap, strong oxidation capacity, high chemical stability, and environmental friendliness [28,29,30]. Under light irradiation, g-C_3_N_4_ generates photogenerated electron–hole (e^−^-h^+^) pairs, thereby producing free radicals to degrade organic pollutants [31]. Although g-C_3_N_4_ has been widely applied in the treatment of refractory wastewater, such as dye and antibiotic wastewater [32,33], its application in the treatment of organic peroxide wastewater has not been reported yet. Therefore, this study prepared g-C_3_N_4_ photocatalyst by thermal polycondensation, systematically investigated its catalytic degradation performance on organic peroxide production wastewater under visible light, and determined the optimal reaction conditions. This work aims to provide an experimental basis and technical reference for the efficient treatment of such wastewater.

## 2. Results

### 2.1. Characterization of g-C_3_N_4_

#### 2.1.1. UV-Vis DRS Characterization of g-C_3_N_4_

**Figure 1:**
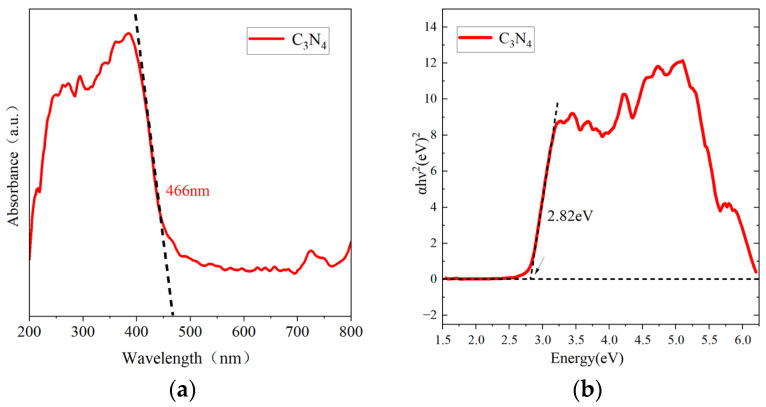
(**a**) UV–visible spectrum; (**b**) diffuse reflection spectrum.

The optical properties of the synthesized catalyst were evaluated via UV-vis diffuse reflectance spectroscopy (UV-vis DRS) across a spectral range of 200–800 nm. As illustrated in Figure 1a, the material exhibited robust light-harvesting capabilities in the ultraviolet region (200–400 nm). Notably, this material still exhibits strong light-harvesting capability in the visible-light region (400–466 nm). To quantify the electronic structure, the optical bandgap energy ($E_{g}$) was derived using the Kubelka–Munk function:

(1) $${\left(ahv\right)}^{\frac{1}{n}}=A\left(hv-Eg\right)$$

where α, h, ν, and A represent the absorption coefficient, Planck’s constant, light frequency, and a proportionality constant, respectively, while n (set to 1/2 for g-C_3_N_4_) corresponds to the semiconductor transition type. By plotting ${\left(\alpha h\nu \right)}^{1/n}$ against photon energy (hν) based on Equation (1), the optical bandgap ($E_{g}$) was determined via linear extrapolation of the absorption edge. As shown in Figure 1b, the intersection with the energy axis yields a bandgap of 2.82 eV. This energy level was sufficient to drive the transition of photogenerated charge carriers, thereby facilitating the formation of electron–hole pairs essential for oxidative degradation processes [34].

#### 2.1.2. SEM and EDS Characterization of g-C_3_N_4_

To characterize the surface features, a Gemini SEM 500 microscope coupled with an EDS detector was employed.

**Figure 2:**
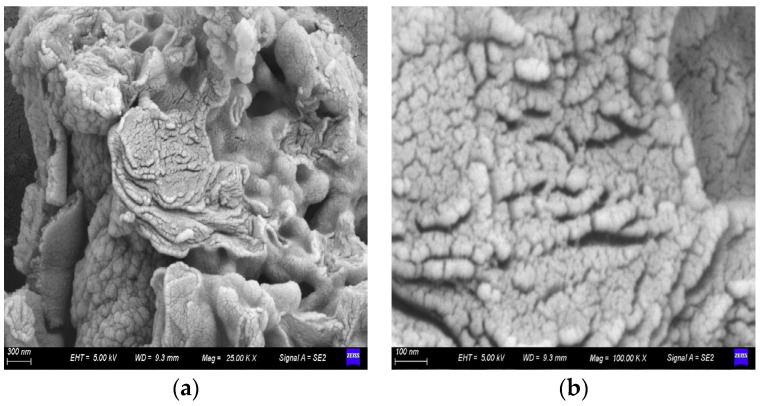
SEM image of photocatalyst: (**a**) ×25 KX; (**b**) ×100 KX.

As shown in Figure 2a, the material exhibited a loose and irregular block morphology, assembled by the stacking of numerous nano-units with distinct internal folding and laminating. Abundant gaps and voids were formed between these structural units. The material possessed a layered structure composed of thin nanosheets (Figure 2b), with irregular and interlaced edges that created plentiful pores and open channels. Its surface was rough with clear textures and wrinkles, which shortened the migration distance of photogenerated carriers, promoted their rapid transfer to the surface for reaction, and thus improved the photocatalytic oxidation rate.

As it is a widely used technique for elemental analysis [35], EDS was employed to investigate the composition of the g-C_3_N_4_ sample.

**Figure 3:**
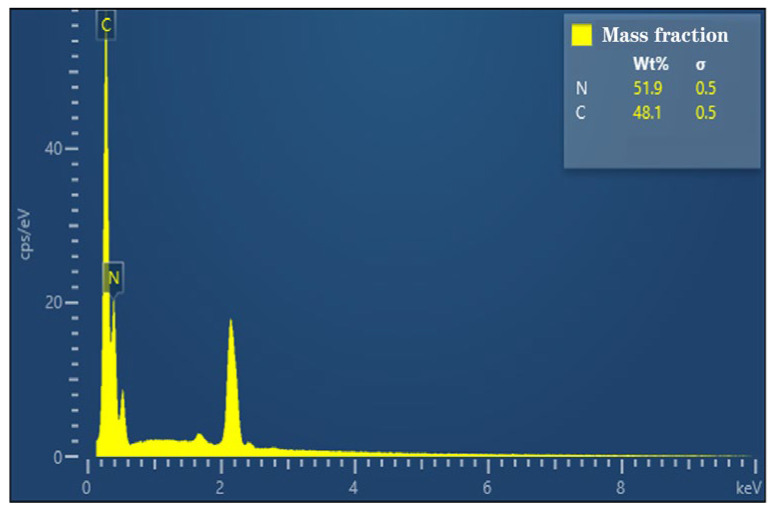
EDS image of photocatalyst.

As shown in Figure 3, the carbon and nitrogen elements were distributed highly uniformly, confirming the successful synthesis of carbon nitride with homogeneous composition. The ideal C/N mass ratio of stoichiometric is 0.643, corresponding to a carbon content of approximately 48.1 wt.% and a nitrogen content of approximately 51.9 wt%. A slight deviation from the theoretical value was observed, which may be attributed to the loss of a small amount of nitrogen in the form of ammonia (NH_3_) during high-temperature calcination, resulting in a relatively higher carbon content in the material [36].

#### 2.1.3. BET Characterization of g-C_3_N_4_

**Figure 4:**
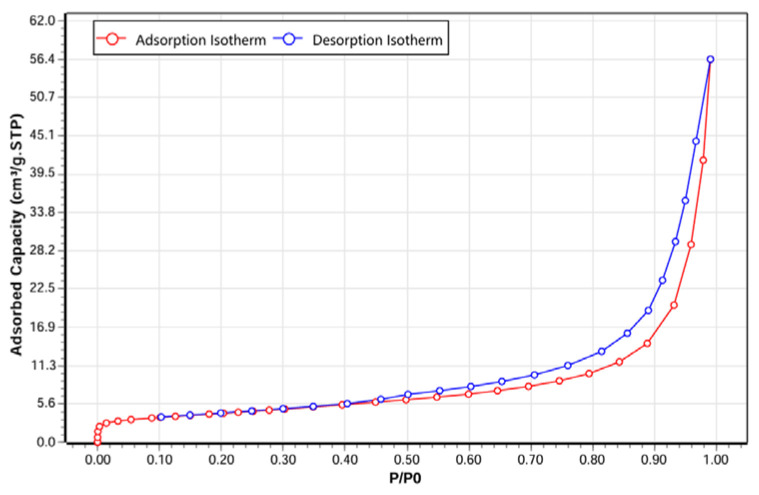
Nitrogen adsorption–desorption isotherm linear plot.

The specific surface area and total pore volume of the prepared g-C_3_N_4_ sample were 15.14 m^2^/g and 0.087 cm^3^/g, respectively (Table 1), which could provide relatively abundant active sites for the photocatalytic reaction. The average pore diameter of the sample was 22.87 nm (Table 1). Meanwhile, the nitrogen adsorption–desorption isotherm of the sample (Figure 4) presented a typical Type IV isotherm with a Type H3 hysteresis loop, indicating that the catalyst was mainly composed of a mesoporous structure and could provide effective diffusion channels for pollutant molecules.

### 2.2. Visible-Light Photocatalytic Oxidation

#### 2.2.1. Effect of g-C_3_N_4_ Dosage

The pH was set to 4.5, which was the pH of the raw water; the light intensity was 1200 W/m^2^, the volume of wastewater used was 200 mL, and the reaction time was 4 h. The influent COD concentration was 14,927 mg/L, and the g-C_3_N_4_ dosage was set at 0.6 g/L, 0.8 g/L, 1.0 g/L, 1.2 g/L, and 1.4 g/L.

**Figure 5:**
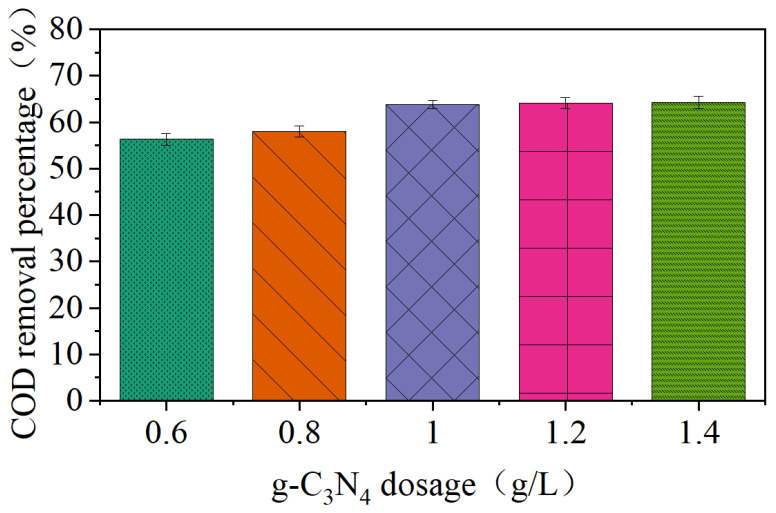
Effect of g-C_3_N_4_ dosage on pollutant removal percentage.

As depicted in Figure 5, the COD removal percentage increased with the g-C_3_N_4_ dosage in the range of 0.8–1.0 g/L but leveled off when the dosage was further increased to 1.4 g/L. Therefore, 1.0 g/L was selected as the optimal catalyst dosage. The increase in g-C_3_N_4_ dosage expands the total surface area of the system, enhancing the rate of photon contact. This drives more valence band electrons to transition to the conduction band, forming a greater number of photogenerated e^−^-h^+^ pairs, which in turn promotes the generation of reactive oxygen species, ultimately enhancing the COD degradation performance [37]. When the dosage of g-C_3_N_4_ exceeded 1.0 g/L, the particles tended to agglomerate and form a dense layer, which caused light refraction and hindered further light penetration into the reaction system. As a result, the COD removal percentage no longer increased [38].

#### 2.2.2. Effect of Light Intensity

The g-C_3_N_4_ dosage was 1.0 g/L, the pH was set to 4.5, the wastewater volume was 200 mL, the influent COD was 14,513 mg/L, and the reaction time was 4 h. Light intensity was systematically varied at 1100 W/m^2^, 1200 W/m^2^, 1300 W/m^2^,1400 W/m^2^ and 1500 W/m^2^.

**Figure 6:**
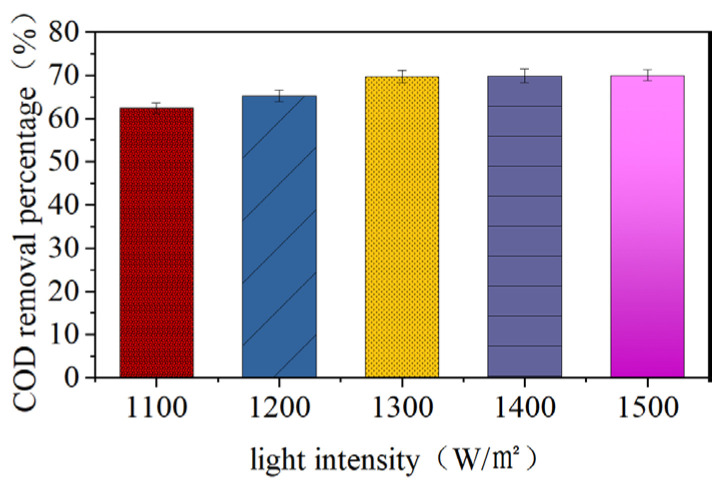
Effect of light intensity on pollutant removal percentage.

As shown in Figure 6, the COD removal percentage increased with rising light intensity from 1100 W/m^2^ to 1300 W/m^2^ but plateaued between 1300 W/m^2^ and 1500 W/m^2^. Based on this observation, an intensity of 1300 was established as the optimal operational parameter. The reason was that within the light intensity range of 1100 to 1300 W/m^2^, the generation rate of photogenerated electron–hole pairs increased linearly with light intensity. Under steady-state conditions, the concentration of effective charge carriers was proportional to the square root of light intensity, which caused continuous generation of reactive oxygen species (ROS) and led to a rapid rise in the COD removal percentage [39]. However, due to the limited number of available active sites on the catalyst surface, once the light intensity reached a saturation level, further increasing the light intensity no longer significantly improved the generation rate of reactive species, and thus the COD removal percentage tended to be stable [40].

#### 2.2.3. Effect of pH

In this study, 200 mL of wastewater with an influent COD concentration of 12,277 mg/L was treated using a catalyst dosage of 1.0 g/L, the light intensity was 1300 W/m^2^, and the reaction time was fixed at 4 h. The pH of the reaction system was adjusted to 3.0, 4.0, 5.0, 6.0, 7.0, and 8.0 using dilute acids (H_2_SO_4_) and bases (NaOH). The optimal reaction pH was determined by measuring the wastewater COD removal percentage as the evaluation index.

**Figure 7:**
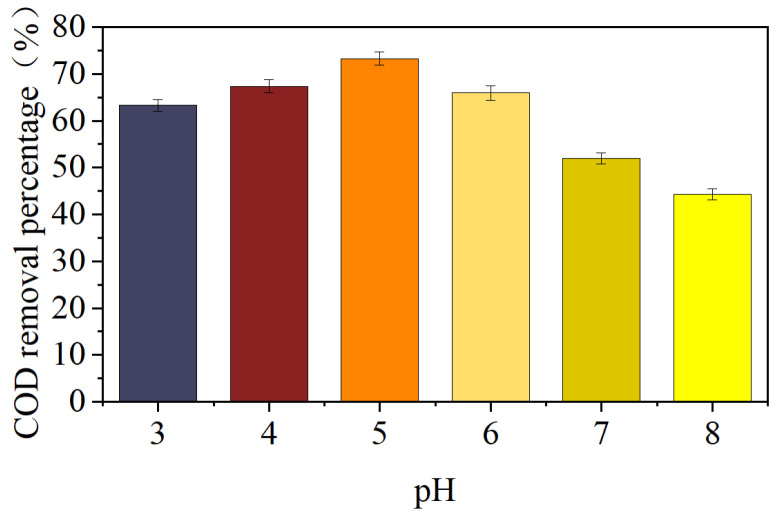
Effect of pH on pollutant removal percentage.

As depicted in Figure 7, as the pH increased from 3 to 5, the COD removal percentage improved, whereas a decline was observed in the range of 5 to 8. Consequently, pH 5 was selected as the optimal condition. The reason was that at a strongly acidic pH below 5, a high concentration of H^+^ captured photogenerated electrons and inhibited the generation of ·O_2_^−^. Meanwhile, it destroyed the catalyst structure, resulting in a decreased degradation rate [41]. When the pH was increased to 5, the separation rate of photogenerated electron–hole pairs was improved, promoting the production of reactive oxygen species (·OH and ·O_2_^−^) and thus enhancing the oxidative degradation of organic pollutants [42]. At a pH above 5, OH^−^ adsorbed onto and occupied the active sites of the catalyst while inhibiting radical generation, ultimately leading to a lower COD removal percentage [43].

#### 2.2.4. Effect of Reaction Duration

The pH was adjusted to 5.0, the wastewater volume was 200 mL, the light intensity was 1300 W/m^2^, the catalyst dosage was set at 1.0 g/L, and the influent COD concentration was 12,453 mg/L. Under these constant conditions, the reaction duration varied at 1, 2, 3, 4, 5, and 6 h, and the appropriate reaction time was selected based on the wastewater COD removal percentage as the evaluation criterion.

**Figure 8:**
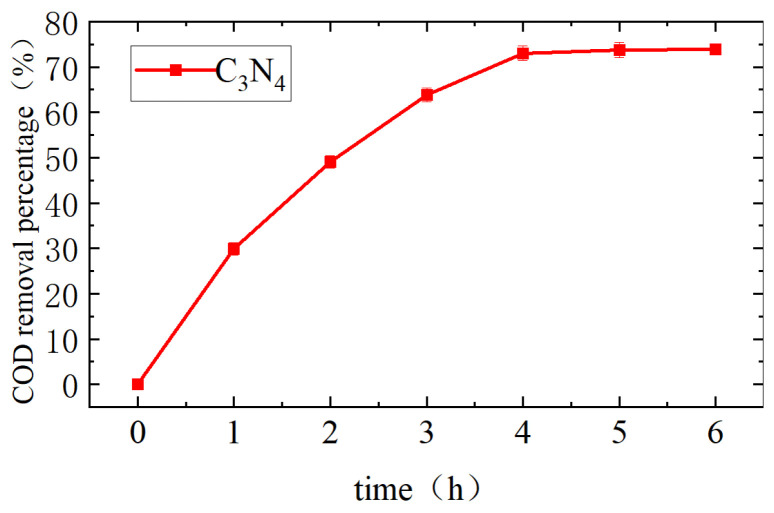
Effect of reaction duration on pollutant removal percentage.

As shown in Figure 8, the COD removal percentage exhibited a steady increase as the reaction time was extended from 1 to 4 h, leveling off in the range of 4–6 h. Consequently, 4 h was selected as the optimal reaction time. The underlying mechanism can be explained as follows: At the initial reaction stage, the high concentration of organic pollutants and abundant active sites on the catalyst favored the prompt generation of reactive oxygen species under visible light irradiation, which effectively oxidized organic contaminants and led to a sharp rise in COD removal percentage. With the reaction proceeding, readily degradable organics were substantially depleted, accompanied by an increased proportion of refractory compounds. Concurrently, the catalytic active sites were occupied by intermediate products, which suppressed the production of reactive species and resulted in a decreased degradation rate [44].

#### 2.2.5. g-C_3_N_4_ Visible Light Photocatalytic Oxidation Kinetics Study

A total of 200 mL of wastewater was adopted with an initial influent COD concentration of 12,453 mg/L. The catalyst dosage was set as 1.2 g/L, and the light intensity was fixed at 1300 W/m^2^. The reaction time was controlled at 1 h, 2 h, 3 h and 4 h to explore the effect of reaction duration on COD removal efficiency by the g-C_3_N_4_ visible-light photocatalytic system. The variation of COD concentration at different reaction times is shown in Table 2. On this basis, zero-order, first-order and second-order kinetic models were used for linear fitting of the degradation process, as shown in Table 3.

According to the fitting results in Table 2 and Table 3, the correlation coefficients R^2^ of the zero-order, first-order and second-order kinetic models were 0.8732, 0.9353 and 0.9878, respectively. The kinetic fitting results showed that the linear correlation coefficient (R^2^ value) was 0.9878, indicating that the second-order kinetic model exhibited the best goodness of fit. In this study, the COD concentration of the organic peroxide production wastewater was as high as >12,000 mg/L. Combined with GC-MS characterization, it was confirmed that aromatic and etheric organic intermediates such as acetophenone, α-methylstyrene, and methyl tert-butyl ether could be generated in the photocatalytic oxidation degradation system. The degradation of high-concentration organic peroxide production wastewater closely followed second-order kinetics. This finding is consistent with the conclusion of Saedi Z et al. [45], who reported that the degradation of high-concentration Congo red and methyl orange dye wastewater tends to follow second-order kinetics.

#### 2.2.6. Effects of Photocatalysis on COD Removal Percentage and Biodegradability

Under the optimized conditions of pH = 5, g-C_3_N_4_ dosage of 1.0 g/L, light intensity of 1300 W/m^2^, reaction time of 4 h, and the influent COD concentration was 14,763 mg/L. The COD and BOD_5_ of the wastewater were determined to calculate its B/C(BOD_5_/COD) ratio; a higher ratio indicates better biodegradability, meaning the wastewater is more suitable for biological treatment. Under optimal conditions, the COD removal percentage of the wastewater system increased from 18% to 72.8% with the addition of g-C_3_N_4_ photocatalyst. This demonstrates that the g-C_3_N_4_ photocatalytic system can significantly improve the treatment performance of organic peroxide-containing wastewater. As shown in Table 4, photocatalytic treatment exhibited a remarkable improvement, achieving a COD removal percentage of 72.8%. This represents an improvement of 37.1% relative to the 35.7% COD removal percentage attained by iron–carbon micro-electrolysis for the same wastewater [21]. In comparison, our photocatalytic system demonstrated a better removal effect than the copper titanate catalyst synthesized by Li et al., which yielded a 58.3% COD removal percentage in treating organic peroxide wastewater [23].

The biodegradability of the wastewater was significantly enhanced following photocatalytic pretreatment with g-C_3_N_4_, evidenced by the rise in the B/C ratio from 0.118 to 0.193. This improvement suggests that the photocatalytic process effectively cleaved recalcitrant macromolecules into simpler, biodegradable intermediates. Consequently, the effluent quality was rendered more compatible with downstream biological treatment systems, validating the efficacy of the g-C_3_N_4_ pretreatment strategy.

### 2.3. Reusability of the Catalyst

To assess the long-term stability and reusability of the g-C_3_N_4_ photocatalyst, four consecutive degradation cycles were performed. The experiments were conducted under optimal operating conditions: 1.0 g/L catalyst loading, pH 5, and an initial COD of 14,367 mg/L in a 200 mL volume. Following each run, the catalyst was separated via centrifugation (2500 rpm), thoroughly washed, and dried before being reintroduced into a fresh wastewater sample for the next cycle.

**Figure 9:**
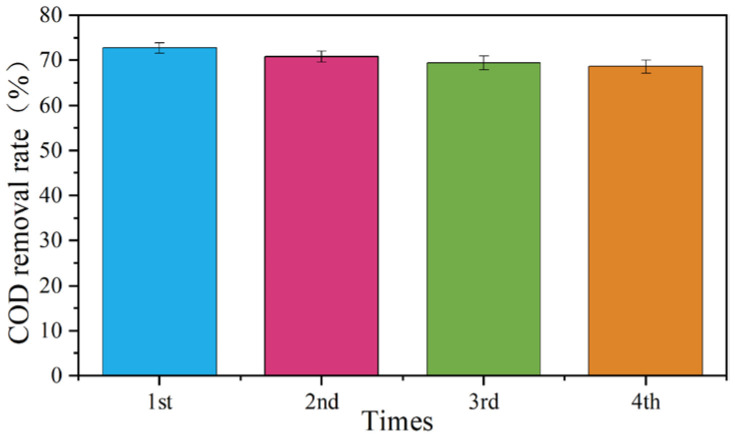
g-C_3_N_4_ photocatalytic repeat reaction removal percentage.

As depicted in Figure 9, the photocatalyst maintained a high degradation rate of 68.23% even after four consecutive cycles, with only a marginal decline of 4.5% from the initial 72. 8% removal percentage. This negligible loss in activity, primarily attributed to minor catalyst loss during recovery, underscores its potential for practical wastewater treatment applications. The synthesized g-C_3_N_4_ demonstrated robust structural stability throughout the recycling tests.

### 2.4. Transformation of Organic Compounds and Reaction Mechanism Analysis

#### 2.4.1. UV-Vis Analysis

UV-vis spectroscopy is an analytical technique for detecting chromophores such as conjugated double bonds and aromatic rings, which is realized by measuring the absorbance of samples at different wavelengths and plotting the absorption spectra [46,47].

**Figure 10:**
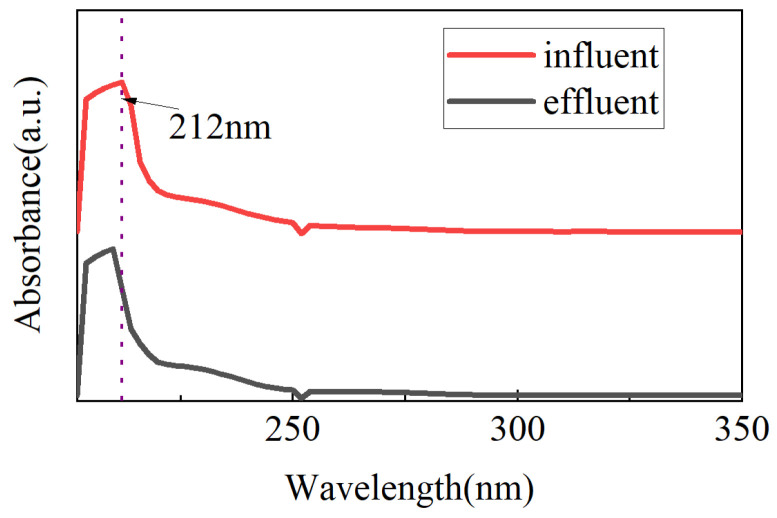
UV-vis of influent and effluent.

UV-vis absorption spectroscopy was performed on the wastewater before and after the photocatalytic reaction. The UV-vis spectra in the range of 205–350 nm are shown in Figure 10.

As shown in Figure 10, the attenuation of the absorption peak at 212 nm, which corresponds to electronic transitions in conjugated systems (e.g., C = C and C = O bonds), confirms the degradation of aromatic and unsaturated organic compounds [48]. Furthermore, the UV-vis spectra remained featureless in other regions, with no emerging peaks detected. This observation suggests that the photocatalytic process did not lead to the accumulation of stable, UV-active intermediate by-products, implying a direct mineralization pathway or the conversion of pollutants into small molecules.

#### 2.4.2. GC–MS Analysis

GC-MS technology combines the high-rate separation capabilities of gas chromatography (GC) with the detection capabilities of mass spectrometry (MS), enabling the effective identification of multiple organic pollutants in wastewater. [49,50]. In this experiment, GC-MS analysis was performed on the organic peroxide production wastewater before and after the photocatalytic reaction.

**Figure 11:**
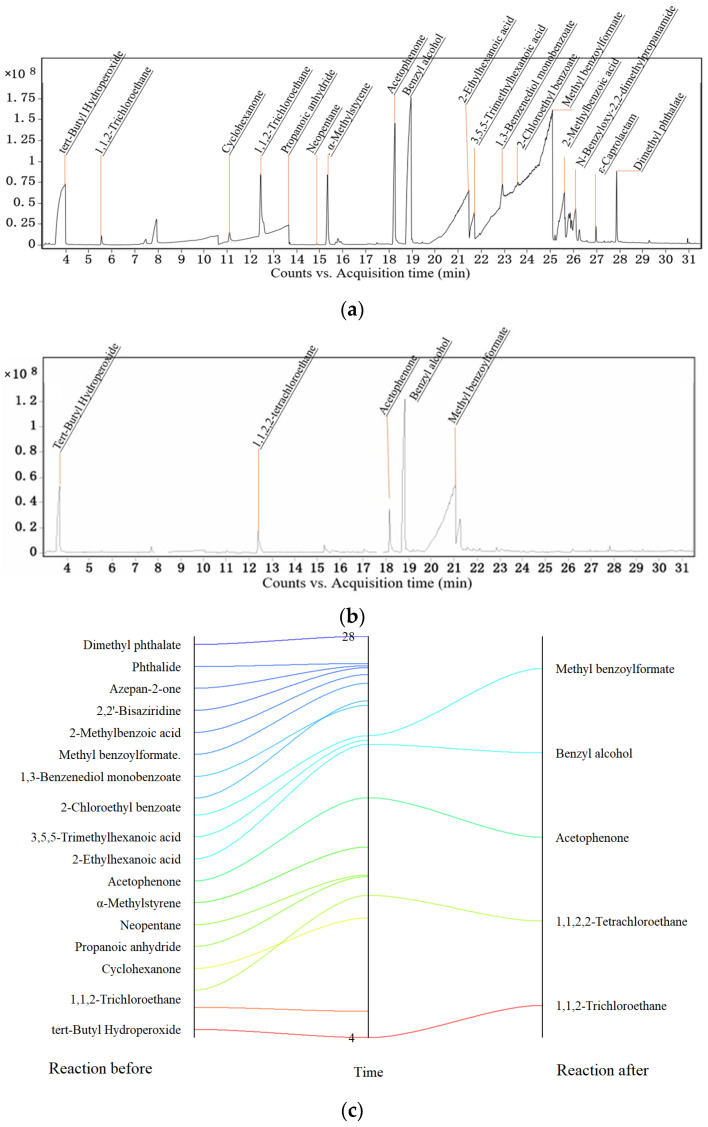
GC-MS spectra of (**a**) influent, (**b**) effluent, and (**c**) compound composition.

By comparing the GC-MS spectra of influent and effluent (Figure 11a,b), the spectra indicate a substantial reduction in contaminant diversity, with hazardous compounds such as 1,1,2-trichloroethane, 2-chloroethyl benzoate, and caprolactam becoming undetectable post-treatment. This confirms the system’s capability to degrade halogenated hydrocarbons, esters and amides. While most organic matter was effectively eliminated, trace amounts of recalcitrant species—including tert-butyl hydroperoxide (TBHP), acetophenone, and 1,1,2,2-tetrachloroethane—persisted in the effluent (Figure 11c). Despite the incomplete removal of these specific intermediates, the overall data demonstrates a robust oxidation performance for most wastewater constituents.

#### 2.4.3. Mechanism Analysis

To elucidate the reaction mechanism, radical trapping experiments were conducted using tert-butanol, AgNO_3_, EDTA-2Na, and chloroform as scavengers for ⋅OH, e^−^, h^+^, and ⋅O_2_^−^, respectively. These tests were performed under optimal operational parameters: 1500 W/m^2^ light intensity, pH 5.0, and a catalyst dosage of 1.2 g/L in a 200 mL reactor.

**Figure 12:**
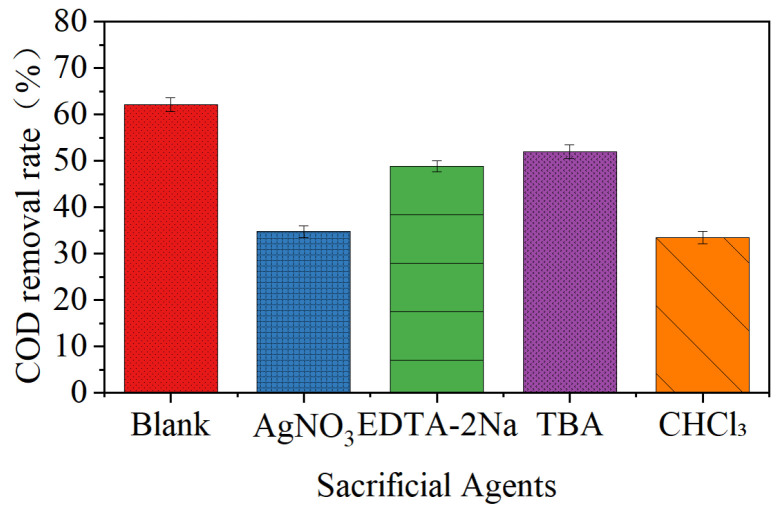
Free radical quenching experiment.

The results are presented in Figure 12; in the absence of scavengers, the blank control achieved a COD removal percentage of 62.10%. Radical quenching experiments revealed that the photocatalytic process was most susceptible to chloroform (CHCl_3_), with the degradation rate plummeting to 33.45% upon its addition. This was followed by AgNO_3_ and EDTA-2Na, which suppressed the removal percentages to 34.72% and 48.81%, respectively. In stark contrast, the introduction of tert-butanol (TBA) resulted in a relatively moderate decline to 51.96%. This distinct inhibitory trend (CHCl_3_ > AgNO_3_ > EDTA-2Na > TBA) directly indicates that the contribution of reactive species follows the order ·O_2_^−^ > e^−^ > h^+^ > ·OH. Consequently, the superoxide radical (·O_2_^−^) and photogenerated electron (e^−^) were identified as the primary active species driving the degradation of organic peroxide production wastewater.

The reactions occurring in the catalytic system are proposed as follows [51,52]:

(2) $$g-C_{3}N_{4}+light\to h^{+}+e^{-}$$

(3) $$h^{+}+{OH}^{-}\to \cdot OH$$

(4) $$e^{-}+O_{2}\to \cdot O_{2}^{-}$$

(5) $$Macromolecules+\cdot OH/\cdot O_{2}^{-}\to h^{+}+Small\;molecules+H_{2}O+CO_{2}$$

Part of the superoxide radicals directly participates in the oxidation of organic compounds, and another part reacts with water to generate hydroxyl radicals for the reaction:

(6) $$\cdot O_{2}^{-}+2H_{2}O\to \cdot OH+{OH}^{-}+O_{2}$$

The oxidation of amides and esters by superoxide radicals (⋅O_2_^−^) mainly proceeds via nucleophilic attack, hydrogen abstraction, and cleavage of C–N and C–O bonds, generating small molecules such as carboxylic acids, and alcohols, which are further mineralized subsequently [53,54]. These active radicals react with pollutants in the organic peroxide production wastewater and decompose amides, esters and other substances [55]. The reaction processes are as follows:

(7) $$R-COO-R^{\prime }+\cdot O_{2}^{-}+H^{+}\to R-COO\cdot +R^{\prime }OH+O_{2}$$

(8) $$R-CO-NH_{2}+\cdot O_{2}^{-}\to R-COOH+O_{2}+NH_{3}$$

Significant changes in the organic composition are observed in Figure 11. The chromatograms reveal the complete removal of 2-chloroethyl benzoate, dimethyl phthalate, and caprolactam at retention times of approximately 23, 27, and 28 min, respectively. These findings align with the reaction pathways proposed in Equations (7) and (8), indicating that the photocatalytic oxidation successfully mineralizes or transforms high-molecular-weight esters and amides into simpler, less toxic aliphatic compounds.

## 3. Materials and Methods

### 3.1. Chemicals and Wastewater

In this study, H_2_SO_4_, NaOH, and melamine were obtained from Tianjin Damao Chemical Reagent Co., Ltd. (Tianjin, China), while tert-butanol, AgNO_3_, EDTA-2Na, and chloroform were supplied by Zhiyuan Chemical Reagent Co., Ltd. (Tianjin, China). All chemical reagents used were of analytical grade.

The experimental water was collected from an auxiliary agent factory in Lanzhou. Samples from different batches appeared with slight variations in water quality, with a pH range of 4.5–6.3, a COD concentration range of 12,300–15,000 mg/L, and a B/C ratio of 0.09–0.11. This wastewater is a refractory, high-concentration organic wastewater.

### 3.2. Preparation of the Catalyst

Graphitic carbon nitride (g-C_3_N_4_) was synthesized via a thermal polycondensation method [56,57]. Specifically, melamine was thoroughly ground, and 10 g of the precursor was placed into a covered ceramic crucible. The sample was then calcined in a muffle furnace at 550 °C for 4 h. Upon completion of the reaction, the furnace was allowed to cool naturally to room temperature. The resulting solid product was collected and ground into a fine powder to obtain the final g-C_3_N_4_.

### 3.3. Properties of Photocatalyst

A UV-vis diffuse reflectance spectrophotometer (UV-3600 Plus, Shimadzu Corporation, Kyoto, Japan) integrated with an integrating sphere was adopted to characterize the light absorption intensity and spectral response range of the prepared catalyst in the 200–800 nm wavelength region, thereby evaluating its optical absorption properties.

A scanning electron microscope (Gemini SEM 500, Carl Zeiss AG, Oberkochen, Germany) was used to observe the surface microstructure and morphology of the catalyst. Before imaging, the catalyst sample was sputter-deposited with a thin gold film to improve its surface conductivity, and all morphological observations were conducted under an accelerating voltage of 12 kV.

### 3.4. Experimental Procedure

To evaluate the photocatalytic performance of g-C_3_N_4_, excluding the contribution of its adsorption, the COD concentration after the catalyst achieved adsorption saturation in the absence of light was set as the initial concentration. Then, 200 mL of wastewater was placed in a photoreactor, and various dosages of the catalyst were tested under visible light (λ ≥ 420 nm) provided by a filtered 300 W xenon lamp (Model H3, Cnlight Co., Ltd., Foshan, China). The experimental setup allowed for precise adjustment of light intensity (measured by SM206). Following centrifugation to remove residual particles, the treated effluent was analyzed for COD removal percentage.

### 3.5. Analytical Methods

To determine the changes in contaminant composition and concentration before and after wastewater treatment, gas chromatography–mass spectrometry (GC-MS 7000C, Agilent Technologies, Santa Clara, CA, USA) [58] and UV-vis absorption spectroscopy (DR5000, Hach, Loveland, CO, USA) [59] were employed.

Standard methods were used for analysis: Biochemical Oxygen Demand (BOD) via dilution inoculation [60]; chemical oxygen demand (COD) via potassium dichromate (Hach DR5000, Loveland, CO, USA) [61]); and pH using a pHS-25 m.

## 4. Conclusions

1. The g-C_3_N_4_ photocatalyst, prepared through thermal polycondensation, demonstrated superior performance in treating organic peroxide wastewater. Specifically, the system achieved a 72.8% reduction in COD and improved the biodegradability index (B/C ratio) from 0.118 to 0.193. These optimal results were obtained after a 4 h reaction under 1300 W/m^2^ irradiation, with a catalyst loading of 1.0 g/L at pH 5. The findings confirm the high photocatalytic activity of g-C_3_N_4_.
2. The photocatalytic degradation mechanism was investigated via radical trapping experiments, which identified superoxide radicals (·O_2_^−^) and photogenerated electrons (e^−^) as the primary reactive species driving the process. In terms of optical properties, the catalyst displayed a band gap of 2.82 eV with an absorption edge at 466 nm, facilitating effective harvesting of visible light. In the recycling tests, the catalyst maintained a high COD removal percentage of 68.23% after four consecutive reaction cycles, demonstrating its excellent stability. Furthermore, UV-vis and GC-MS characterizations demonstrated the system’s capability to decompose complex organics, including esters, amides, and carboxylic acids. Despite a minor decline in activity, recycling tests confirmed the catalyst’s robust stability, suggesting its potential as a viable solution for treating organic peroxide production wastewater.

## Figures and Tables

**Table 1:** Structural characteristics of g-C_3_N_4_.

Sample	Specific Surface Area/(m^2^·g^−1^)	Pore Volume/(cm^3^·g^−1^)	Average Pore Diameter/nm
g-C_3_N_4_	15.14	0.087	22.87

**Table 2:** Relationship between COD concentration and reaction time.

Reaction Duration (h)	Influent COD C_0_ (mg/L)	Effluent COD C_t_ (mg/L)	C_0_ − C_t_	ln(C_0_/C_t_)	1/C_t_ − 1/C_0_
0	12,453	12,453	0	0	0
1	12,453	8731	3722	0.36	3.42 × 10^−5^
2	12,453	6333	6119	0.68	7.76 × 10^−5^
3	12,453	5753	6700	0.77	9.35 × 10^−5^
4	12,453	5013	7439	0.91	0.000119

**Table 3:** Kinetic model fitting parameters for COD degradation.

Kinetic Model	Linear Fitting Equation	Correlation Coefficient R^2^	Rate Constant k
Zero-order reaction	y = 1785 t + 1224	0.8732	1785 mg·L^−1^·h^−1^
First-order reaction	y = 0.2237 t + 0.093	0.9353	0.2237 h^−1^
Second-order reaction	y = 2.98 × 10^−5^ t + 5.38 × 10^−6^	0.9878	2.98 × 10^−5^ L·mg ^−1^·h^−1^

**Table 4:** Performance comparison of organic peroxide wastewater treatment methods.

Process	COD Concentration of Raw Water (mg/L)	Experimental Conditions	Performance	Reference
g-C_3_N_4_ photocatalytic oxidation	14,763	g-C_3_N_4_ dosage of 1.0 g/L, light intensity of 1300 W/m^2^, reaction time of 4 h	The removal of COD was 72.8%	This study
Iron–carbon micro-electrolysis	15,712	Iron filing dosage 30.5 g/L, Fe-C mass ratio 1.01, reaction time 122.8 min, pH = 3.1	The COD removal reached 35.7%	[21]
CuTiO_3_ photocatalytic oxidation	12,900	CuTiO_3_ dosage of 1.0 g/L, light intensity of 1300 W/m^2^, reaction time of 4 h	The COD removal reached 58.3%	[23]

## Data Availability

All data acquired and analyzed in the present research are contained within this paper and can be obtained upon request.
